# Commerson’s dolphin population structure: evidence for female phylopatry and male dispersal

**DOI:** 10.1038/s41598-022-26192-0

**Published:** 2022-12-23

**Authors:** Cristian Alberto Durante, Rocio Loizaga, Gregory R. McCracken, Enrique Alberto Crespo, Daniel E. Ruzzante

**Affiliations:** 1 Laboratorio de Mamíferos Marinos, Centro Para El Estudio de Sistemas Marinos (CESIMAR) - CONICET, Bv. Brown 2915, U9120ACD Puerto Madryn, Chubut, Argentina; 2 Universidad Nacional del Comahue, San Carlos de Bariloche, Rio Negro Argentina; 3 Department of Biology, Dalhousie University, Halifax, NS Canada

**Keywords:** Ecology, Genetics

## Abstract

A key in species conservation is understanding the amount and distribution of genetic diversity and how environmental changes that occurred in the recent past may have influenced current patterns of population structure. Commerson’s dolphin, *Cephalorhynchus commersonii*, has two subspecies, one of which is endemic to South America (*C. commersonii commersonii*) and little is known about its population genetics. Our objective was to investigate the population genetics of this subspecies throughout its distribution. Using 70 skin samples and information available in GenBank, 308 mitochondrial DNA sequences and 28 species-specific microsatellites were analyzed. The species presented low genetic diversity when compared to other dolphin species, but was consistent with other species within the genus. Strong population structure based on mitochondrial DNA was exhibited throughout its entire distribution, a pattern consistent with female philopatry. However, this pattern was not detected when using microsatellites, suggesting male-mediated gene flow. Demographic tests suggested a population expansion beginning approximately 15,000 years ago, after the Last Glacial Maximum. In a climate change scenario, we recommended considering each sampling location as an independent population management unit in order to evaluate the impact of possible environmental changes on the distribution of genetic information within the species.

## Introduction

Understanding population structure is vital for studying the ecology of endemic species and planning or designing conservation strategies^1^. As we improve our understanding of the structuring of natural populations, it becomes essential that researchers also focus on understanding the processes underlying these patterns^2^. Most species are divided into populations, even in apparently continuous environments such as the marine environment, where barriers to gene flow are not always clear and can be associated with currents, temperature, salinity or primary productivity^3–6^.

Despite their broad range and their dispersal capabilities, marine mammals usually exhibit strong population structure at some geographic scale^2,7–10^. Populations may respond differently to a common threat^11^. Their susceptibility to changes will largely depend on genetic variability, i.e. their capacity to cope with environmental changes^12,13^. The largest threats to marine mammals currently include global warming^14^, and other anthropogenic impacts, especially fishery bycatch, resource overfishing, oil exploration, and pollution^15,16^. Predicting how populations will respond to these threats is complex and may depend on factors that shape their distribution patterns (such as prey availability) and their genetic diversity^17^. However, distribution of current genetic diversity also reflects the influence of long-term historical processes. By examining environmental changes that took place over the course of the Pleistocene and Holocene (including climate change), we gain insight into how similar factors will impact contemporary populations^18^. The Last Glacial Maximum (LGM) at the end of the Pleistocene is one of the largest global environmental changes and had significant impact on the genetic diversity of many natural populations of top predators^19^. After the LGM, many populations of marine mammals showed demographic expansion both in the northern and southern hemispheres, such as narwhals *Monodon monoceros*^20^, belugas *Delphinapterus leucas*^21^, bowhead whales *Balaena mysticetus*^22^, South American fur seal *Arctocephalus australis*^23^, South American sea lion *Otaria flavescens*^24,25^, southern right whale *Eubalaena australis*^26^, dusky dolphin *Lagenorhynchus obscurus*^27^, and spectacled porpoise *Phocoena dioptrica*^28^. These historical climatic fluctuations, characterized by periods of contraction and retraction, have been suggested to be the primary driving factor behind the origin and radiation of some species from the southern hemisphere, e.g., those that are involved in the genus *Cephalorhynchus*^29^.

The genus *Cephalorhynchus* includes four dolphin species widely distributed in cool temperate waters of the Southern Hemisphere, with each species endemic to a different region^30^. Among them, Commerson’s dolphin (*Cephalorhynchus commersonii),* has the largest distribution and comprises two subspecies: *C. commersonii commersonii* (South America) and *C. commersonii kerguelenssi* (Kerguelen Islands), which differ from each other both morphologically and genetically^29,31^. Along the southeastern coast of South America, the species is distributed between 40° and 56° S, including Strait of Magellan and the Falkland (Malvinas) Islands^32,33^. It is found frequently in shelf waters (< 200 m deep) and near shore (< 60 km from the Argentine coast and < 25 km from Falkland (Malvinas) Islands), where the continental shelf is wide and flat, there are large tidal cycles, and the waters are relatively cold as they are influenced largely by the Malvinas Current^33–39^. This species is present in different coastal habitats, with the highest densities in areas in close proximity to river mouths^40^.

The population structure of Commerson’s dolphins is poorly known, with existing information primarily focused on the southern range of the species. There, some degree of genetic differentiation at regional scale was detected using mitochondrial markers suggesting site fidelity by females^41,42^. However, there is still a gap of information regarding their northern distribution and contemporary genetic variation. Increasing the sampling effort will lead to a better understanding of how genetic diversity is distributed throughout the species range. Also, the information obtained here is crucial for the development of effective and sustainable management plans for the species^43^. To achieve these goals, we used mitochondrial and microsatellite DNA variability to investigate the population genetic diversity and structure of Commerson’s dolphin throughout their distribution in South America.

## Materials and methods

### Study area, sample collection, and DNA extraction

**Figure 1:**
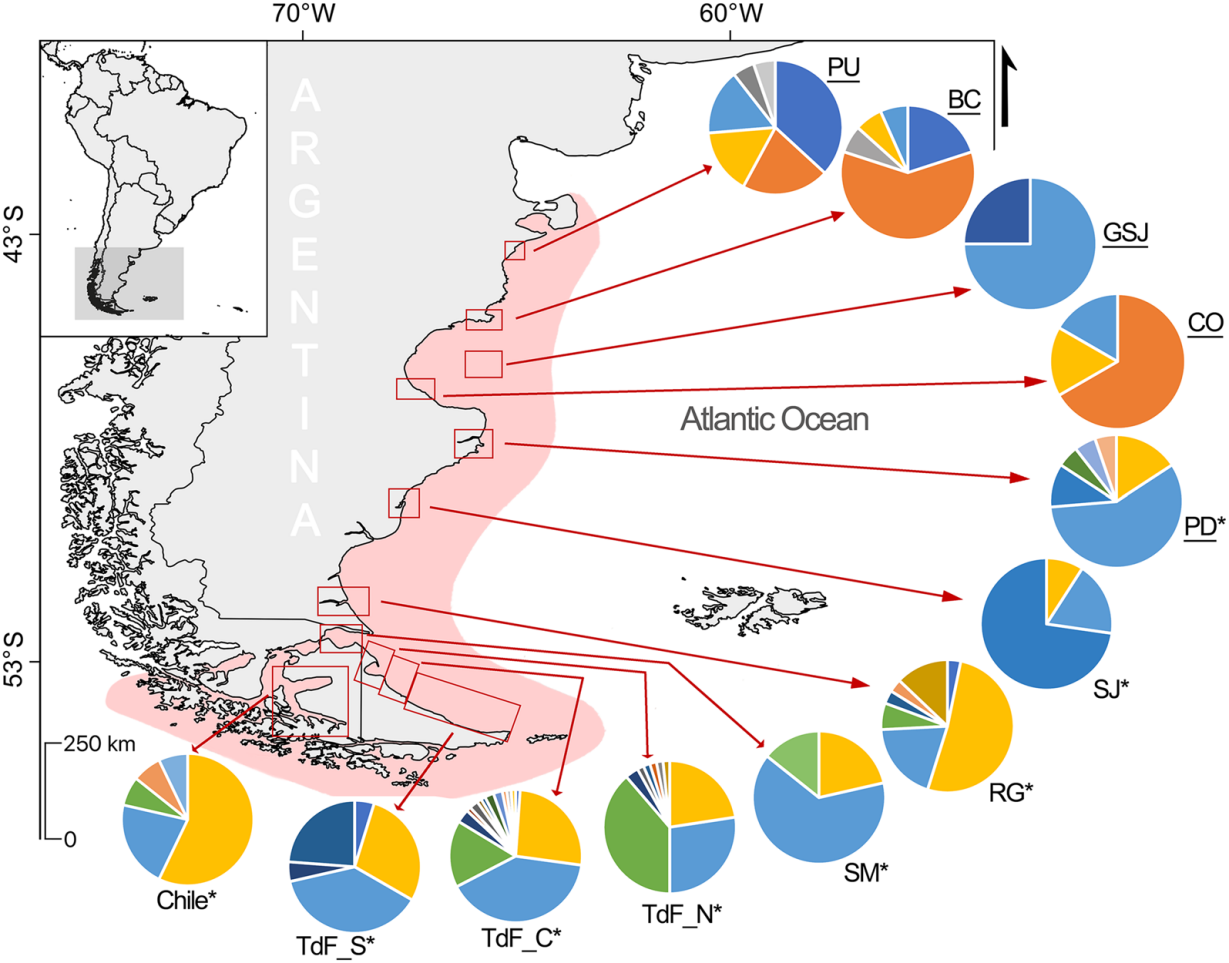
Study area showing *Cephalorhynchus commersonii* distribution in red shadow. Sampling sites underlined correspond to present study, whereas those with asterisk (*) indicate the localities for the mitochondrial DNA control region sequences from Cipriano et al.^41^ and Pimper et al.^42^. PU = Playa Unión, BC = Bahía Camarones, CO = Caleta Olivia, GSJ = Golfo San José, PD = Puerto Deseado, SJ = San Julián, RG = Río Gallegos, SM = Strait of Magellan, TdF = Tierra del Fuego (N = North, C = Center, S = South). Pie charts represent the respective frequency of mitochondrial DNA control region haplotypes from each sampling location. The map was created in QGIS 3.12 (https://www.qgis.org/es/site/), whereas the final figure edition was generated using Photopea online graphics editor (https://www.photopea.com/).

Tissue samples from 70 *Cephalorhynchus commersonii* were collected between 1999 and 2019 from five localities along the Patagonian coast: Playa Unión (43° 16′ S, 65° 02′W), Bahía Camarones (45° 2′ S, 65° 34′ W), Caleta Olivia (46° 26′S, 67° 30′W), Golfo San Jorge (46° 03′ S, 65° 59′ W), and Puerto Deseado (47° 47′ S , 65° 48′ W; Fig. 1). Of these, eight samples were from stranded individuals, eight from incidental capture, and the remaining fifty four were obtained via biopsy skin sampling^44^. All methods are reported in accordance with ARRIVE guidelines and were carried out in agreement with relevant guidelines and regulations. Also, sampling was approved under permits Nº41/2009, Nº002/2009, Nº23/07 N°001/10, Nº178/07, Nº001/19, Nº93/15, Disposición Nº8 and Nº16 awarded by Dirección de Fauna y Flora Silvestre, Subsecretaria de Recursos Naturales, Ministerio de Industria, Agricultura y Ganadería, Subsecretaría de Conservación y Áreas Protegidas, Secretaría de Turismo, Dirección de Fauna Silvestre, Consejo Agrario Provincial and Administración de Parques Naturales. To cover the entire distribution of the species in South America, 253 mitochondrial DNA (mtDNA) control region sequences available in GenBank (accession numbers are presented in Supplementary 1 Table S1, see Pimper et al.^42^ and Cipriano et al.^41^) were included for further analysis, increasing the sampling location to the south of our sampling area (Fig. 1).

Total genomic DNA was extracted following a glass milk protocol modified from Elphinstone et al.^45^ using a Perkin Elmer Multiprobe II Plus Liquid Handling System (Perkin Elmer, Waltham, MA). Before extraction, tissue samples were digested at 55ºC for 2 days, adding 4 μl of Proteinase K (20 mg/ml, New England BioLabs, NEB) approximately every 8 h. DNA quality and quantity were evaluated by electrophoresis on 1% agarose gel. Sex of the individuals sampled via skin biopsy was identified molecularly following Bérubé and Palsbøll^46^ method for the amplification of ZFX and ZFY regions.

### Molecular protocol

#### Mitochondrial DNA

The mtDNA control region was amplified using primers MTCRf (5′-TTCCCCGGTCTTGTAAACC) and MTCRr (5′-TTTTCAGTGTCTTGCTTT)^47^. The reaction was performed in a 20 μl final volume including: 0.5 units of TSG; 2.5 mM MgCl_2_, 200 μM dNTPs, 10 mM buffer (Tris–HCl, pH 8.4), 0.3 μM of each primer and 1 μl of DNA template. The PCR profile was: 3 min at 93 °C; then 30 cycles of 1 min at 92 °C, 1 min at 48 °C and 1 min at 72 °C; then a final extension phase of 5 min at 72 °C. Each PCR product was purified with enzymatic PCR cleanup at 37 °C for 30 min and then at 80 °C for 20 min, prior reaction using: 0.025 μl of Exonuclease I (NEB), 0.25 μl Antarctic Phosphatase (NEB), 2.5 μl Antarctic Phosphatase buffer (NEB), and 7.225 μl of H_2_O. For all samples, a target fragment of ~ 900 bp was sequenced at MacrogenUSA (Rockville, MD), followed by visual checks and alignment on MEGA7^48^.

#### Microsatellites

A single sample with the highest quality and quantity DNA was selected for shotgun sequencing and microsatellite design by means of visualization on 1% agarose gel stained with GelGreen (BioTium Fremont, CA). Our sequencing library was developed using 1 ng of genomic DNA following the Illumina Nextera XT Sample Preparation Kit protocol (Illumina, San Diego, California)^49^. Briefly, the protocol consisted of tagmentation genomic DNA, followed by PCR amplification. Purification of the PCR products was conducted using in-house made speedbeads follow Faircloth, et al.^50^. DNA library sequencing was performed using a MiSeq Benchtop Sequencer (Illumina, San Diego, California). Forward read output data from the MiSeq were imputed into Msatcommander 1.0.8-beta^51^ for the detection of microsatellite loci, with final product size, including primers and flanks, between 50 and 120 bp. A total of 34,973 microsatellites and 5017 sets of primers were identified following this criterion, and 70 were chosen at random for amplification/testing. Amplification was carried out in two multiplex PCR reaction (35 microsatellites each one) per individual. Further details on the molecular protocol to primer amplification are available in Ruzzante et al.^49^ and references therein. Summarized, this step consisted of diluting the multiplex PCR products and indexing them via a second PCR. PCR products were then pooled in equal proportion and subsequently cleaned via speedbeads. After quantification, the library was diluted to 15 pM and sequenced in a single direction using an Illumina MiSeq Benchtop Sequencer (Illumina, San Diego, CA). Post-sequencing data were automatically genotyped using Megasat^52^ followed by manual verification.

### Analyses

#### Mitochondrial DNA

Haplotype and nucleotide diversity^53^ were estimated with DNAsp v5^54^, considering each sampling location as a separate population. The analysis of molecular variance (AMOVA) based on F_ST_ (using haplotypes frequencies) and Φ_ST_ (using genetic distances with Kimura 2-parameter algorithm) was performed in Arlequin^55,56^, and significance was tested using 10,000 permutations. The substitution model (Kimura 2-parameter) was selected under Akaike Information Criterion (AIC) generated with jModelTest v2.1.10^57^. These analyses were tested under two scenarios: using all datasets and a data subset that includes sampling sites with microsatellite information (PU, BC, CO, PD) avoiding related individuals (see next section: “Microsatellites”). Gene flow among populations was estimated using genetic structure measures (Φ_ST_) in Arlequin^58^. A haplotype network applying the median-joining algorithm and default parameters was built in Network 5.0.1.0^59^. Demographic tests were explored through the Fu neutrality test^60^ and mismatch distribution analysis^61^ using Arlequin 3.5.2.2^58^. A Mantel test was performed in Genalex 6^62^ to examine isolation by distance through the use of linearized Φst^63^. BEAUTi v1.8.3 and BEAST v1.8.3^64^ were used for Bayesian skyline plot reconstruction, assuming a piecewise-linear Bayesian skyline tree prior. The substitution model selected was Hasegawa–Kishino–Yano (HKY) based on AIC generated with jModelTest v2.1.10^57^. In addition, we used a lognormal relaxed clock rate (uncorrelated) with a mutation rate for the control region of 6.3 × 10^–7^ s/s/year derived for *Lagenorhynchus obscurus*^65^. Ninety million iterations of the MCMC (Markov Chain Monte Carlo) were applied, discarding the first 9,000,000 steps as “burn in” and sampling every 1000 iterations. Finally, convergence was checked through the effective sample size (ESS > 200) and a Bayesian skyline plot was built, both using Tracer v1.6^66^.

#### Microsatellites

Potential null alleles were assessed using Microchecker 2.2.3^67^. Tests for Hardy–Weinberg equilibrium (HWE) and linkage disequilibrium (LD) between pairs of loci were conducted in Arlequin 3.5.2.2^58^, applying 1,000,000 Markov chain steps (MCMC) with 100,000 dememorization steps and 30,000 permutations, respectively; *p* values were adjusted using false Discovery rate adjustment^68^. Duplicates among the samples were tested using Genalex 6^62^, removing one individual from each resampled pair. Allelic richness (A_R_) for each location were estimated with FSTAT 2.9.3^69^, and Arlequin 3.5.2.2^58^ was used to estimate observed (Ho) and expected heterozygosity (He). Effective population size was estimated with LDNe^70^. Population structure was estimated through AMOVA using Arlequin 3.5.2.2^58^. Also, hierarchical population structure analysis was performed in Structure 2.3.4^71^ under the admixture model, using 5,000,000 MCMC, 1,500,000 burn-in steps, and 10 replicates for each genetic cluster (K = 1–8). The Evanno method^72^ implemented in Structure Harvester^73^ was used to estimate the most likely K. Both genetic differentiation tests were conducted using two datasets: first using all samples and, second a data subset without related individuals. To account for filial relatedness, *Queller and Goodnight 1989 estimator*^74^ was applied using Genalex 6^62^. Those individuals with a relatedness coefficient ≥ 0.5 (first-order relationship, such as parents and offspring or full-siblings) were removed. Contemporary gene flow among sampling locations was estimated using BayesAss Version 3^75^, with a run setting of 150,000,000 iterations, 10% burn-in, and the rest of parameters as default. The model convergence was checked through the effective sample size (ESS > 200) using Tracer v1.6^66^. Finally, a Mantel test^63^ was performed in Genalex 6^62^ to examine isolation by distance among sampling sites using linearized F_ST_ values.

## Results

### mtDNA

We amplified the mtDNA control region in 55 of 70 individuals sampled, with the per individual sequence length ranging from 326 to 698 bp; the shortest sequences belong to the oldest samples. Three duplicate individuals (identified with microsatellite loci) were removed from the analysis. We used a 423 bp consensus sequence to align all sequences available in GenBank. From a total of 308 sequences used for population analyses, we found 26 polymorphic sites, defining 27 distinct haplotypes distributed throughout the species distribution (Fig. 1). Five of these haplotypes are novel for the species (Table 1, Supplementary 1 Table S1). Overall haplotype diversity was high (h = 0.801 ± 0.014), while nucleotide diversity was moderate (π = 0.00477 ± 0.00298) (Table 1).

**Table 1:** Summary of mitochondrial DNA control region (423 bp) data of Commerson’s dolphin (*Cephalorhynchus commersonii*) for each sampling site (including data from Cipriano et al.^41^ and Pimper et al.^42^). N = sample size, Hap = Number of haplotypes, h = Haplotype diversity, π = Nucleotide diversity, CG = Cytosine and Guanine percentage, Ts = transitions, Tv = transversions, I = indels, TdF = Tierra del Fuego.

Sampling sites	N	Hap	Nº variant sites	h	π (%)	CG (%)	Ts	Tv	I
Playa Unión	19	6	7	0.8070 ± 0.0587	0.5336 ± 0.3429	34.87	7	–	–
Bahía Camarones	15	5	13	0.6286 ± 0.1253	0.6233 ± 0.3950	34.93	13	–	–
Caleta Olivia	6	3	3	0.6000 ± 0.2152	0.2848 ± 0.2418	34.95	3	–	–
Golfo San Jorge	4	2	1	0.5000 ± 0.2652	0.1184 ± 0.1467	35.17	1	–	–
Puerto Deseado	19	6	11	0.6550 ± 0.1115	0.5748 ± 0.3641	35.13	11	–	–
San Julián	11	3	3	0.4727 ± 0.1617	0.1898 ± 0.1666	35.36	3	–	–
Río Gallegos	31	7	8	0.6946 ± 0.0738	0.3256 ± 0.2293	34.82	8	–	–
Strait of Magellan	14	3	3	0.5604 ± 0.1245	0.2973 ± 0.2236	35.02	3	–	–
TdF_North	62	9	8	0.7335 ± 0.0302	0.3703 ± 0.2483	35.00	8	–	–
TdF_Center	92	14	15	0.7484 ± 0.0299	0.4298 ± 0.2767	35.01	15	–	–
TdF_South	21	5	6	0.7476 ± 0.0517	0.3232 ± 0.2317	35.01	6	–	–
Chile	14	5	6	0.6593 ± 0.1227	0.3077 ± 0.2293	34.82	6	–	–
Overall	308	27	26	0.801 ± 0.014	0.477 ± 0.298	34.99	26	–	–

AMOVA showed significant genetic differentiation among all sampling areas (populations defined a priori), F_ST_ = 0.139 and Φ_ST_ = 0.194, suggesting female philopatry (Table 2). Differences among populations are significant and represent almost 20% of variability. Furthermore, most of the pairwise Φ_ST_ values were significant (Table 3) and migration rates estimated using this genetic structure measure were mostly less than five effective migrants (Supplementary 1 Table S2). Data subset without related individuals (n = 42), also, showed significant differentiation based on both genetic structure measures (Supplementary 2 Table S1, S2). Finally, the Mantel test indicated a significant isolation by distance model (R^2^ = 0.14; *p* = 0.03; Supplementary 1 Fig. S1).

**Table 2:** AMOVA statistics for mitochondrial DNA control region sequences from Commerson’s dolphin (*Cephalorhynchus commersonii*) distribution along Southwestern Atlantic Ocean.

Analysis	Source of variation	*df*	Sum of squares	Variance components	Percentage of variation	Statistics	*p* value (α = 0.05)
Haplotype frequencies	Among populations	11	18.58	0.057	13.88	F_ST_ = 0.139	< 0.001
	Within populations	296	104.42	0.353	86.12		
	Total	307	123.00	0.410			
Distance method: Kimura 2-parameter	Among populations	11	62.00	0.204	19.40	Φ_ST_ = 0.194	< 0.001
	Within populations	296	250.53	0.846	80.60		
	Total	307	312.53	1.050

**Table 3:** Genetic differentiation among pairwise populations of Commerson’s dolphin (*Cephalorhynchus commersonii*) using: **a-** mitochondrial DNA control region (423 bp) of all distribution (F_ST_ values are reported below the diagonal, whereas Φ_ST_ values are reported above the diagonal). **b-** 28 microsatellites loci in 50 individuals. Significant values (α = 0.05) are in bold font. PU = Playa Unión, BC = Bahía Camarones, CO = Caleta Olivia, GSJ = Golfo San José, PD = Puerto Deseado, SJ = San Julián, RG = Río Gallegos, SM = Strait of Magellan, TdF = Tierra del Fuego.

		Distance method: Kimura 2-parameter											
**a-**		PU	BC	CO	GSJ	PD	SJ	RG	SM	TdF_north	TdF_center	TdF_south	Chile
Computing conventional F-Statistics from haplotype frequencies	PU		0.07342	0.11690	− 0.00863	**0.09665**	**0.30346**	**0.32741**	**0.12913**	**0.35441**	**0.22208**	**0.15104**	**0.31648**
	BC	0.07589		− 0.06462	0.07637	**0.11545**	**0.33607**	**0.43053**	**0.22189**	**0.44813**	**0.32560**	**0.26751**	**0.39281**
	CO	0.10688	− 0.06520		0.28073	0.07380	**0.48071**	**0.43530**	0.23968	**0.41604**	**0.25952**	**0.27461**	**0.43770**
	GSJ	**0.20462**	**0.38120**	0.36283		− 0.01629	**0.39766**	**0.44597**	0.12612	**0.41184**	**0.22371**	0.19362	**0.49769**
	PD	**0.17276**	**0.32438**	**0.27706**	− 0.04581		**0.17586**	**0.22744**	− 0.01215	**0.26198**	**0.11388**	0.04079	**0.20555**
	SJ	**0.31104**	**0.43230**	**0.45108**	**0.44211**	**0.28607**		**0.52765**	**0.36473**	**0.49352**	**0.35449**	**0.38888**	**0.56999**
	RG	**0.14645**	**0.29619**	**0.25069**	**0.25826**	**0.16183**	**0.33883**		**0.17764**	**0.07661**	**0.04921**	**0.11364**	− 0.03129
	SM	**0.20239**	**0.36886**	**0.32800**	− 0.03408	− 0.02437	**0.39859**	**0.17083**		**0.20848**	0.04024	− 0.00215	**0.18890**
	TdF_north	**0.16784**	**0.28385**	**0.24892**	**0.17181**	**0.13316**	**0.31245**	**0.10931**	**0.14945**		**0.04113**	**0.15048**	0.05843
	TdF_center	**0.13152**	**0.26326**	**0.21559**	0.05623	0.02952	**0.28071**	**0.06823**	0.03381	**0.03274**		0.01918	0.03470
	TdF_south	**0.11421**	**0.26876**	**0.22287**	0.08251	0.04454	**0.30541**	**0.06392**	0.05278	**0.10377**	0.02546		**0.12696**
	Chile	**0.15944**	**0.32070**	**0.26886**	0.27976	**0.16374**	**0.37108**	− 0.03169	**0.17560**	**0.10673**	**0.05838**	0.06620	
**b-**													
Number of different alleles	BC	0.02501											
	CO	0.04152	− 0.00069										
	PD	**0.0896**	**0.04230**	− 0.01505									

**Figure 2:**
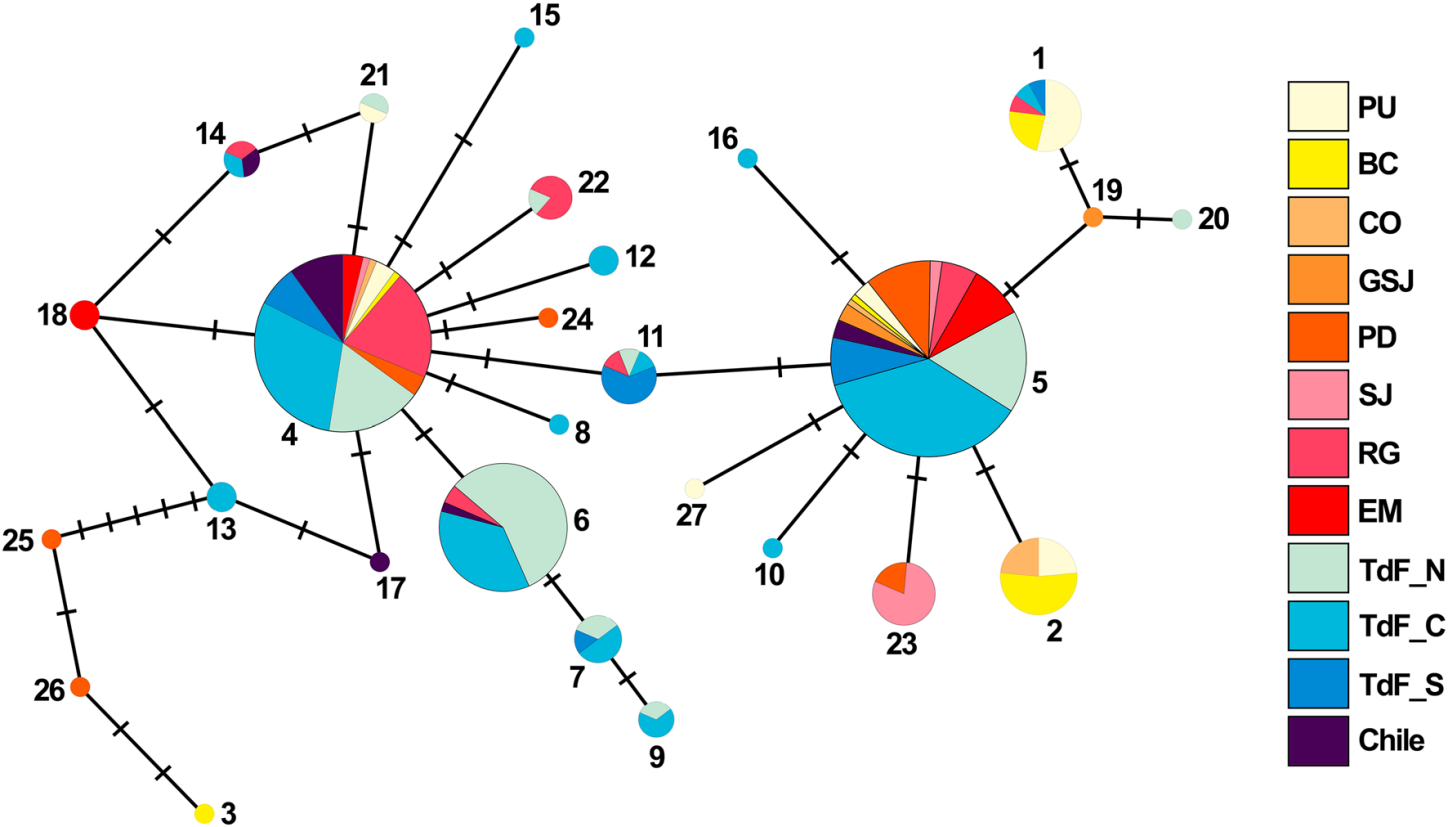
Haplotype network of *Cephalorhynchus commersonii* mitochondrial DNA control region sequences built using the median-joining algorithm (N = 308, 423 bp). The size of the circles is proportional to haplotype frequency, and the dashes between them represent one mutational step away from the next. PU = Playa Unión, BC = Bahía Camarones, CO = Caleta Olivia, GSJ = Golfo San José, PD = Puerto Deseado, SJ = San Julián, RG = Río Gallegos, SM = Strait of Magellan, TdF = Tierra del Fuego.

The minimum spanning network reflects the geographic distribution of the different haplotypes with a star-like configuration suggesting demographic expansion (Fig. 2). Haplotypes H5 and H4 were the most frequent (H5 = 32.48%, H4 = 25.72%). Only four haplotypes were new, singletons and unique to the four northern populations (Playa Unión, Bahía Camarones, Caleta Olivia, Golfo San Jorge), while 23 were shared among populations.

**Figure 3:**
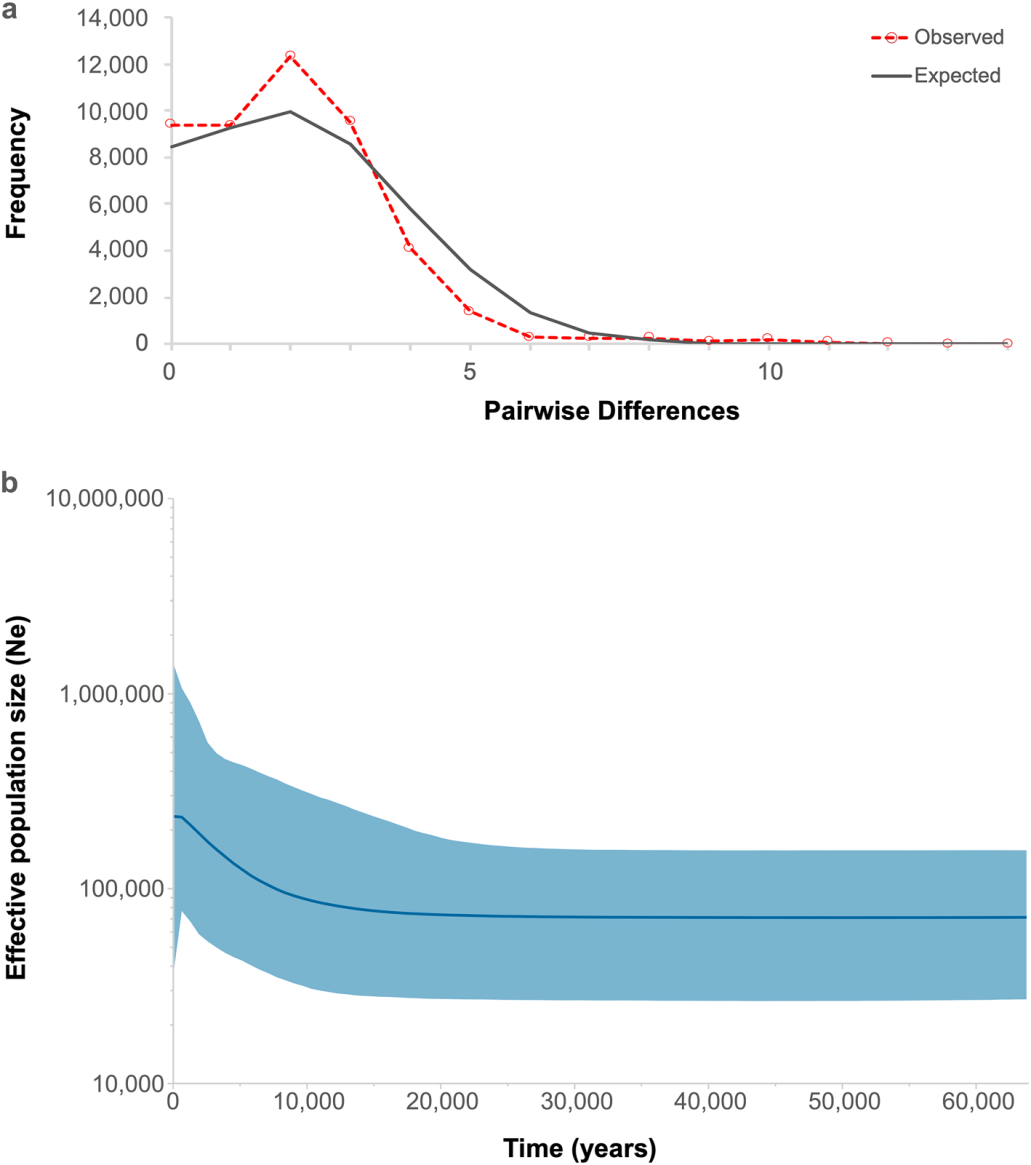
(**a**)—Mismatch distribution analysis according to a population expansion scenario of Commerson’s dolphin (*Cephalorhynchus commersonii*). The observed number of differences are given as a red dashed line, and those expected under a population expansion model are given as a black solid line. (**b**)—Bayesian Skyline plot representing historical demographic trends of Commerson’s dolphin population. Estimates of effective population size (Ne) means are joined by a solid line; whereas the colored area corresponds to the credibility interval based on 95% highest posterior density interval.

Neutrality tests were performed with all population samples combined. Fu’s Fs-values were large, negative, and significant for the entire population (Fu’s Fs = − 13,622, *p* = 0.002, α = 0.02), suggesting a population expansion. Also, the mismatch distribution showed a unimodal distribution suggesting a recent population expansion event (SSD = 0.004, *p* = 0.515; Harpending's raggedness index r = 0.025, *p* = 0.693). Furthermore, Bayesian skyline reconstruction also revealed a recent demographic expansion around 15,000 years ago (Fig. 3).

### Microsatellites

From the 70 microsatellites/primer sets tested, 30 polymorphic loci were amplified successfully in 50 individuals from four sampling locations only (Playa Unión = 17; Bahía Camarones = 13; Caleta Olivia = 6; Puerto Deseado = 14). No evidence of linkage disequilibrium was detected, nor were null alleles detected. Two loci showed significant departure from Hardy–Weinberg equilibrium after false Discovery rate adjustment and were removed from the analysis. Moreover, three individuals were eliminated following their identification as duplicates. Summary statistics for the remaining 28 microsatellites loci in the four populations from north-central Patagonia are presented in Supplementary 1 Table S3, whereas genetic diversity within populations is summarized in Table 4. A total of 100 alleles were detected across all loci, 16 of those alleles were private and all four populations exhibited private alleles. Microsatellite locus CCo8 was the most variable with 15 alleles, followed by CCo52 with 8 alleles. Allelic richness (*A*_*R*_) per locus, which is independent of sample size, ranged from 1.132 to 8.003 and the highest mean over loci *A*_*R*_ was found in Caleta Olivia (3.053). Average Ho and He ranged from 0.315 and 0.320 to 0.348 and 0.341, respectively. No significant difference was found in *A*_*R*_, Ho and He among populations (Kruskal–Wallis test, *df* = 3, *p* > 0.05).

**Table 4:** Summary statistics of Commerson’s dolphin (*Cephalorhynchus commersonii*) for 28 microsatellites loci by sampling site. N = Sample Size, Na = Number of Alleles, AR = Allelic Richness (based on min. sample size of 7 diploid individuals), Ne = Effective population size, I = Information Index, Ho = Observed Heterozygosity, He = Expected, and F = Fixation Index. Mean and standard deviation (SD) are reported, except for Ne where the 95% confidence interval [CI] is displayed.

Sampling site	N	Na	A_*R*_	Ne	I	Ho	He	F
**Playa Unión**								
Mean	16.857	2.929	2.684	176.9	0.609	0.348	0.341	− 0.021
SE	0.067	0.445	1.559	[33.7-inf]	0.103	0.056	0.051	0.034
**Bahía Camarones**								
Mean	12.929	2.786	2.666	31.2	0.605	0.329	0.338	0.044
SE	0.050	0.350	1.476	[12.6-inf]	0.098	0.055	0.049	0.046
**Caleta Olivia**								
Mean	6.000	2.393	3.053	354.2	0.557	0.315	0.320	0.010
SE	0.000	0.274	1.311	[7.4-inf]	0.097	0.057	0.052	0.054
**Puerto Deseado**								
Mean	14.000	2.679	2.759	24.7	0.582	0.339	0.323	0.005
SE	0.000	0.326	1.317	[11.8–130.0]	0.098	0.056	0.050	0.040

**Figure 4:**
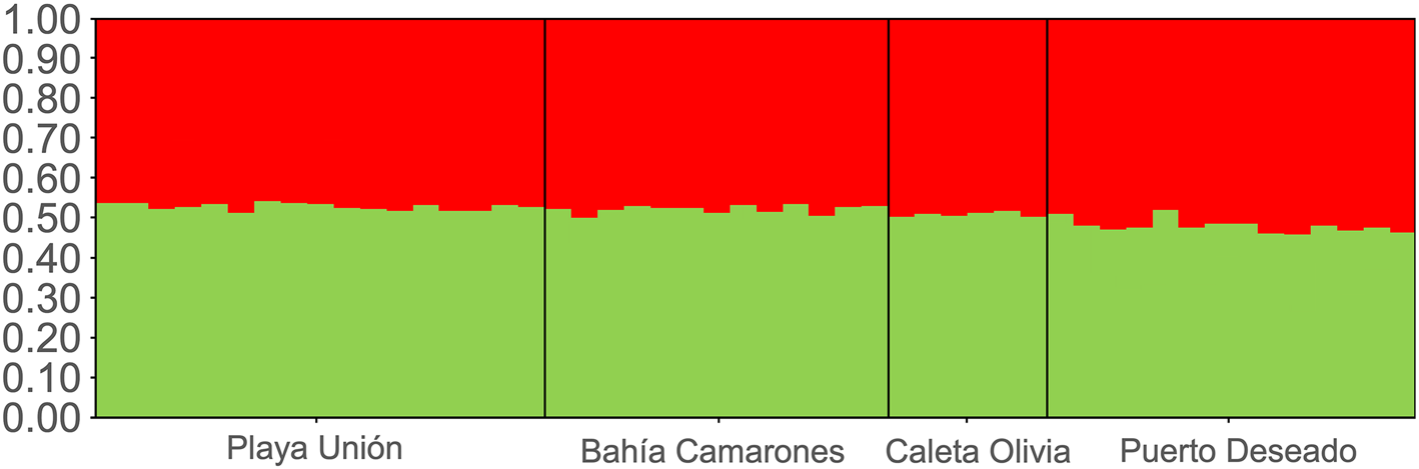
Hierarchical STRUCTURE analysis of Commerson’s dolphin (*Cephalorhynchus commersonii*) from the four sampling regions based on 28 microsatellite loci and 50 individuals. Each individual is represented by a vertical line, which is partitioned into K colored segments, where each colored segment length is proportional to the individual's estimate membership coefficient.

Although AMOVA analysis showed evidence of genetic structure (F_ST_ = 0.032, *p* = 0.023); this result is based in only two small significant F_ST_ values between the two most geographically distanced populations: Playa Unión (43° S) and Bahía Camarones (45° S) with Puerto Deseado (47°S) (Table 3). Evanno et al.^72^ method indicated K = 2 as the most likely scenario to Structure assignment test, however, high variance, low Delta K values, and the fact that no individual was 100% assigned to a single genetic cluster, suggest only one panmictic population (Fig. 4, Supplementary 1 Table S4, S5 and Fig. S2). Both genetic differentiation analyses using the data subset without related individuals (n = 39) showed a single population (AMOVA: F_ST_ = 0.012, *p* = 0.316; Supplementary 2 Table S3, S4 and Fig. S1, S2). Using a Mantel test, the correlation was significant, supporting the presence of isolation by distance between regions (R^2^ = 0.75; *p* = 0.04; Supplementary 1 Fig. S3). Finally, contemporary migration rates were less than 0.1 between populations (Supplementary 1 Table S6).

## Discussion

In marine mammal population genetic studies, a common assumption is that the species under study represent a single genetic population, with likely some degree of gene flow between predefined "populations"^76^. However, coastal odontocetes (like the species under study) are prone to population fragmentation and isolation, even in the short-term and along the contiguous coastline without physical barriers, which is probably due to natal fidelity^77,78^. Through the use of mtDNA and species-specific microsatellites we analyzed the population genetics of Commerson’s dolphins to understand how its genetic information is distributed along their range in southern South America. Our results showed that Commerson's dolphin represents a single panmictic population, with a strong female philopatry along the entire distribution based on mtDNA data and gene flow mediated by males at least in the northern of its distribution where microsatellite data could be analyzed.

Genetic structure analysis based in mtDNA and nuclear DNA markers exhibited contrasting patterns. Considering the mtDNA control region analysis a strong genetic structure among Commerson’s dolphins sampling locations was detected, suggesting a strong female philopatry which is not biased by kinship relationships as when kin pairs were detected one member of the pair was eliminated from the analysis. Female dispersal was low (nM < 5), which is consistent with significant breaks in dispersal. In contrast, no structure among northern populations was detected using microsatellite data, supporting the idea of male-mediated gene flow at least in the northern region of Patagonian continental shelf. In genetic populations studies of mammals, is common to assume the existence of sex-biased dispersal behavior, which is based on female philopatric behavior due to the requirements needed to breed and parental care^18,79^. Also, males dispersal is expected to improve reproductive success and avoid inbreeding depression^18^. Although it is best known in terrestrial mammals^79^, several studies with marine mammals, especially with cetaceans, support this hypothesis showing male-mediated gene flow^18,80–82^. However, it does not appear to be a common pattern for the species in the genus *Cephalorhynchus*. Based on mitochondrial markers, Heaviside’s dolphin, *C. heavisidii*, did not show evidence of population structure^83^, whereas Chilean dolphin, *C. eutropia*, showed genetic differentiation only through microsatellite analysis^84^. In contrast, Hector’s dolphin, *C. hectori*, displayed significant genetic differentiation and limited gene flow among populations using mtDNA and microsatellites^85,86^. Particularly with Commerson’s dolphin from South America, previous genetic analysis of mtDNA population structure found high levels of genetic differentiation indicating a strong site fidelity by females^41,42^. This was also confirmed in the present study, which covers the entire Commerson’s dolphin distribution in South America and extensive survey of microsatellite data, not reported before.

The genetic variability estimated with both molecular markers is distributed homogeneously suggesting, also, a single genetic population of Commerson’s dolphin. Furthermore, those estimated through mtDNA is similar to previously reported for the species in the south of its distribution (between 47 and 56° S) and for the genus^41,42,78,87^.

A combination of high h (> 0.5) and low π (< 0.005) is characteristic of species/populations that suffered a population bottleneck followed by a rapid demographic expansion and the consequent accumulation of mutations^88^. In this context, the Commerson´s dolphin population of the coast of Argentina might have experienced periods of population expansion and contraction coupled with glaciation events that occurred during the Pleistocene. Thus, population growth and geographic expansion occurred, likely with rapid diversification of haplotypes with minimum nucleotide differences. The haplotype network in a star-shape coincides with the results of the paired differences curve (mismatch distributions) and Fu values, suggesting recent population expansion for the species.

Other marine mammals from the region also exhibited recent demographic expansion^23,24,26–28^ likely associated with the LGM and sea ice retreat, around 15,000 years ago (as it is reported here based on Bayesian skyline reconstruction analysis). Habitat changes including changes in the distribution and abundance of prey are likely to have taken place during this period. These changes in primary productivity and relative abundance of prey played a key role in the history of some coastal dolphin populations. An example is dusky dolphin historic demography which was coupled with fluctuations in anchovy populations, its main prey^17^. Marine mammals were favored by these changes, leading to a sudden demographic expansion along the coast of the Southwestern Atlantic Ocean^29,89^.

The hypothesis of *Cephalorhynchus* radiation, based on the west-wind drift mechanism, suggests that Commerson’s dolphin is one of the latest species to establish its current distribution, after setting up the New Zealand population^29^. Once established in South America, the species appears to have expanded northward across the south-western Atlantic Ocean coasts. As can be visualized in the haplotype network (Fig. 2) and in the haplotype composition of each sampling site (Fig. 1), the majority of haplotypes were concentrated in the south of Argentina but many of them were found throughout almost the entire distribution. Among these haplotypes, the ancestral haplotypes (H4 and H5)^41,42^ are geographically spread throughout the entire distribution along southwestern Atlantic ocean. Therefore, historical changes in the habitat added to the recent radiation of the species with a rapid demographic expansion, is likely to have led to the absence of population genetic structure.

Population expansion from the southern to the northern ranges, with strong female philopatry and contemporary male-mediated gene flow, can produce the isolation-by-distance pattern (IBD) observed in our data^90^. However, while it is possible that the IBD mechanism is not the most appropriate model to represent the complexities of natural populations, it is a useful hypothesis for testing different scenarios. Commerson´s dolphin migration rates differed between marker type. The low sample size used with microsatellites is likely a factor contributing to the failure to identify recent migrants with BAYESASS^91^. Longer movements among far away locations may also occur more frequently than between nearby locations, e.g., between the northern and southern populations, where the southern populations act as a source (where the density of individuals is the highest) and the northern as a sink (with the lowest density)^39^. Although the species' range has not been estimated, it was reported individual movements of up to 250 km in distance^34^. This range of movement is higher than the home range estimated for other species of the genus (< 60 km)^30^, and it may explain the lack of a clear pattern of population structure, such as "stepping-stone model" suggested for Hector's dolphins population^85^. Consequently, the Commerson´s dolphin population on the coast of Argentina might respond to a meta-population model. Therefore, it is necessary to increase the analysis with nuclear markers analysis using samples from southern localities to have a better understanding of the population structure.

The condition of panmixia does not necessarily mean that the species should be considered as a single management unit. If indeed the species is structured according to a metapopulation, any impact at the local level could disrupt the dynamic of the populations and generate isolation between them. Taking into account the effects caused by past climatic fluctuations and in a current climate change scenario, alterations in sea surface temperature could produce changes in the habitat and affect the population structure of the species. To assess whether the pattern of genetic differentiation in the species conforms to a metapopulation model, an increased sampling effort in terms of both sample size and geographic coverage is recommended. The analysis of molecular markers in the present study is geographically uneven, with the analysis of autosomal markers restricted to the northern part of the species distribution. Hence, based on the precautionary principle, we recommend that the sampling areas analyzed here should be considered as single management units, where future evaluations and technical reports consider them independently.

## Supplementary Information

Supplementary Information 1.

Supplementary Information 2.

## Data Availability

All the data generated or analyzed during this study are included in this published article (and its Supplementary Information file). Molecular markers information is available in Genbank (https://www.ncbi.nlm.nih.gov/genbank/) under accession numbers ON776962-ON777036. The dataset analyzed in the study is available upon request by contacting the corresponding author.
