# Supplementary material for: Commerson’s dolphin population structure: evidence for female phylopatry and male dispersal
Source: Sci Rep. 2022 Dec 23;12:22219. doi: 10.1038/s41598-022-26192-0 (PMC9789068; doi:10.1038/s41598-022-26192-0)
Supplement: Supplementary file 1 — Supplementary Information 1. [file 41598_2022_26192_MOESM1_ESM.pdf]

COMMERSON`S DOLPHIN POPULATION STRUCTURE: EVIDENCE FOR FEMALE PHYLOPATRY AND MALE DISPERSAL

Cristian Alberto Durante, Rocio Loizaga, Gregory R. McCracken, Enrique Alberto Crespo, Daniel E. Ruzzante.

Supplementary material 1

Table S1: Position of the 26 polymorphic sites defining 27 distinct haplotypes (h) within 423 bp of the mtDNA control region of Commerson's dolphin (*Cephalorhynchus commersonii*), including frequency by haplotype (Freq) and GenBank accession number.

H	Freq	3	42	51	101	113	124	136	173	207	213	216	219	233	237	250	258	263	271	325	338	359	360	362	377	393	420	GenBank number
1	13	C	A	T	T	C	G	T	C	G	T	A	C	A	T	T	A	C	G	C	T	A	T	C	A	G	C	HM368541.1/HM749893.1
2	17	·	G	·	·	·	·	·	·	·	·	·	·	·	C	·	·	·	·	T	·	·	·	·	·	·	·	ON777032 (Present study)
3	1	·	G	·	C	·	·	·	·	A	C	·	T	·	C	C	G	T	·	T	·	·	·	T	·	·	T	ON777033 (Present study)
4	80	·	G	·	·	·	·	·	·	A	·	·	·	·	C	·	·	·	A	·	·	·	·	·	·	·	·	AF393538.2/HM749884.1/HM368540.1
5	101	·	G	·	·	·	·	·	·	·	·	·	·	·	C	·	·	·	·	·	·	·	·	·	·	·	·	AF393537.2/HM749881.1/HM368538.1
6	42	·	G	C	·	·	·	·	·	A	·	·	·	·	C	·	·	·	A	·	·	·	·	·	·	·	·	AF393539.2/HM748989.1
7	6	·	G	C	·	·	·	·	·	A	·	·	·	·	C	·	·	·	A	·	·	·	C	·	·	·	·	AF393540.2
8	1	·	G	·	·	·	·	·	·	A	·	·	·	·	C	·	·	·	A	·	C	·	·	·	·	·	·	AF393543.2
9	3	·	G	C	·	·	·	C	·	A	·	·	·	·	C	·	·	·	A	·	·	·	C	·	·	·	·	HM368539.1
10	1	·	G	·	·	·	A	·	·	·	·	·	·	·	C	·	·	·	·	·	·	·	·	·	·	·	·	HM368542.1
11	8	·	G	·	·	·	·	·	·	A	·	·	·	·	C	·	·	·	·	·	·	·	·	·	·	·	·	HM368543.1/HM749894.1
12	2	·	G	·	·	·	·	·	·	A	·	G	·	·	C	·	·	·	A	·	·	·	·	·	·	·	·	HM368544.1
13	2	·	G	·	·	·	·	·	·	A	·	·	·	·	C	·	·	·	A	T	·	·	·	·	·	·	T	HM368545.1
14	3	·	G	·	·	·	·	·	T	A	·	·	·	·	C	·	·	·	A	·	·	·	·	·	·	·	T	HM368546.1/HM749892.1
15	1	·	G	·	·	T	·	·	·	A	·	·	·	G	C	·	·	·	A	·	·	·	·	·	·	·	·	HM368550.1
16	1	·	G	·	·	·	·	·	·	·	·	·	·	·	C	·	·	·	·	·	·	·	·	·	·	·	T	HM368551.1
17	1	·	G	·	·	·	·	·	·	A	·	·	·	·	C	·	·	·	A	T	·	·	·	·	·	·	·	HM368548.1
18	2	·	G	·	·	·	·	·	·	A	·	·	·	·	C	·	·	·	A	·	·	·	·	·	·	·	T	HM749888.1
19	1	·	·	·	·	·	·	·	·	·	·	·	·	·	C	·	·	·	·	·	·	·	·	·	·	·	·	ON777034 (Present study)
20	1	·	·	·	·	·	·	·	·	·	·	·	·	·	C	·	·	·	A	·	·	·	·	·	·	·	·	HM368547.1
21	2	·	G	·	·	·	·	·	T	A	·	·	·	·	C	·	·	·	A	·	·	·	·	·	·	·	·	HM368549.1
22	5	T	G	·	·	·	·	·	·	A	·	·	·	·	C	·	·	·	A	·	·	·	·	·	·	·	·	HM368552.1/HM749887.1
23	10	·	G	·	·	·	·	·	·	·	·	·	·	·	C	·	·	·	·	·	·	G	·	·	·	·	·	HM749886.1
24	1	·	G	·	·	·	·	·	·	A	·	·	·	·	C	·	·	·	A	·	·	·	·	·	G	·	·	ON777035 (Present study)
25	1	·	G	·	C	·	·	·	·	A	C	·	T	·	C	·	G	·	·	T	·	·	·	·	·	·	T	HM749890.1
26	1	·	G	·	C	·	·	·	·	A	C	·	T	·	C	·	G	·	·	T	·	·	·	T	·	·	T	HM749891.1
27	1	·	G	·	·	·	·	·	·	·	·	·	·	·	C	·	·	·	·	·	·	·	·	·	·	A	·	ON777036 (Present study)

Table S2: Number of migrants among populations estimated from genetic structure measures (Φ_{ST}) for all sampling locations. PU = Playa Unión, BC = Bahía Camarones, CO = Caleta Olivia, GSJ = Golfo San José, PD = Puerto Deseado, SJ = San Julián, RG = Río Gallegos, EM = Strait of Magellan, TdF = Tierra del Fuego.

Sampling locations	PU	BC	CO	GSJ	PD	SJ	RG	SM	TdF_north	TdF_center	TdF_south	Chile
PU	-											
BC	6.30978	-										
CO	3.77731	inf	-									
GSJ	Inf	6.04674	1.28109	-								
PD	4.67336	3.83070	6.27492	inf	-							
SJ	1.14767	0.98780	0.54013	0.75737	2.34315	-						
RG	1.02714	0.66136	0.64863	0.62114	1.69841	0.44759	-					
SM	3.37210	1.75340	1.58607	3.46447	Inf	0.87087	2.31463	-				
TdF_north	0.90684	0.61576	0.70180	0.71407	1.40852	0.51314	6.02630	1.89832	-			
TdF_center	1.75144	1.03564	1.42664	1.73500	3.89059	0.91047	9.66005	11.92600	11.65681	-		
TdF_south	2.81031	1.36911	1.32077	2.08232	11.75700	0.78575	3.89998	Inf	2.82259	25.57556	-	
Chile	1.07986	0.77288	0.64233	0.50465	1.93253	0.37721	inf	2.14692	8.05671	13.90993	3.43825	-

Table S3: Characteristics of 70 microsatellites loci designed for Commerson's dolphin (*Cephalorhynchus commersonii*) including locus name, primer sequence, annealing temperature (Ta), repeat motif, number of observed alleles (Na), observed heterozygosity (Ho), expected heterozygosity (He), number of successfully amplified individuals (N), and microsatellite classification (P = Polymorphic, M = Monomorphic, NA = Not amplified).

LOCUS	Primer Squence 5'-3'	Ta	Repeat motif	Na	He	Ho	N	Size Range	
CCOM1	F:TGTGAGGACAGAAGCAGAGG<br>R:GCAGCATGGCATCTTCAAAC	59	(AG)10	2.000	0.449	0.460	53	60-62	P
CCOM2	F:GGAAGGGAGGTAGAGAACGAC<br>R:CTTTAGGGCTGGAGGTTCAAAG	60	(AG)10	-	-	-			M
CCOM3	F:GAAACATGGATGCCCTGCC<br>R:TGTCTTTCTCTGCCCACCC	60	(AG)13	-	-	-			M
CCOM4	F:TGGGAAGTATTGTCTCACCAC<br>R:GGCCCGATGTCAAGATTTCTC	59	(AG)10	2.250	0.421	0.349	53	65-69	P
CCOM5	F:GCCTAACAGACTTGACACACC<br>R:GCGTGCTTGCGTGTCTAC	60	(AC)14	4.250	0.645	0.631	53	65-73	P
CCOM6	F:TTGGGTAGCAGAACTGGACG<br>R:ACAATAGTGAGAGGCCCGC	60	(AC)10	-	-	-	-		NA
CCOM7	F:TTTAGGTCACATAGGCACATCC<br>R:GGCAGCTCTTGTATGCGTG	59	(AC)12	-	-	-	-		NA
CCOM8	F:CAGATGCCAGATGAGTACGTG<br>R:CTTCTTAGACCAGCCTGTGAC	59	(AC)15	9.750	0.853	1.000	53	59-99	P
CCOM9	F:CCCGCAAAGCCCATACCC<br>R:GGGTTATAGGTGTAGTGTTGGG	60	(AC)13	-	-	-	-		NA
CCOM10	F:ACAGGTAATCTCCCAAGGAAGG<br>R:AAGGTGCTAAGACCCAAACG	59	(AG)9	-	-	-			M
CCOM11	F:GGCCCTGGAGAATGAAAGAC<br>R:AGGGAGATCAGAAGAGTTGGG	59	(AG)8	1.250	0.017	0.018	53	74-76	P
CCOM12	F:GTGTCACAGAGGAATGTCAACC<br>R:ACGCAGACACACGTTAAGC	60	(AC)13	2.500	0.483	0.423	53	73-77	P
CCOM13	F:GAAGCCAGCCTGAGTGTATG<br>R:TGCTTCCAGGAATTTCTCTCC	59	(AC)8	-	-	-	-		NA
CCOM14	F:AGAGTGTGTGAGTGCGGAC<br>R:CTCTCCCGAGCAGAGTGAC	60	(AC)13	-	-	-	-		NA
CCOM15	F:TCCTGCACTTCTCACACCTC<br>R:GGGTGGAGGAATGGACATTG	60	(AC)13	3.000	0.331	0.363	53	77-89	P
CCOM16	F:TAACCCAACCCACAGCATCC<br>R:GGAGAGAGTTCAAGCAAGTGTC	60	(AC)14	4.250	0.588	0.577	53	78-96	P
CCOM17	F:AGTCTGTCTCCATGGCTCC<br>R:TGGCCCTAAATGCAATGACTG	59	(AG)10	2.000	0.345	0.365	53	76-78	P
CCOM18	F:CCCATGCAACAGTCATCACC<br>R:TCCTCTCTTGATAACCCTGCTG	60	(AC)10	-	-	-			M
CCOM19	F:TCGTGGGAGGTAGGGTAAGG<br>R:GGGAGCTAAGTCAGAGAAATGC	60	(AC)10	1.500	0.027	0.028	53	79-83	P
CCOM20	F:GACCCTCATCGTGCTTCAAG<br>R:ACAGCCAACACCAGAGTACG	60	(AC)10	1.500	0.050	0.054	53	79-81	P
CCOM21	F:GTACCTGAAACACTGCACGC<br>R:TTTCTGCCAGATACCCAAGTC	59	(AC)17	2.750	0.302	0.113	53	78-92	P
CCOM22	F:TCTCAGTTCAGGGTGTACGG<br>R:CATGGACAGATACACAGTGGG	59	(AC)8	1.750	0.122	0.107	53	80-86	P
CCOM23	F:CGGTGTTGTTGAGATTCAGGG<br>R:CATGGGCAGACAGAGACATC	59	(AC)18	-	-	-	-		NA
CCOM24	F:GCAAAGACCTTAGTTCTGCAC<br>R:ACATTTCATACCACCACGCAG	59	(AC)15	3.750	0.636	0.712	53	83-91	P

CCOM25	F:GCAGTGTGGGCAGAATGTG<br>R:GATACCCGGCTCCTTTCCTC	60	(AC)11	1.750	0.091	0.098	52	91-93	P
CCOM26	F:GTGGAGAGCAGCAATTTGAAG<br>R:GCACAGCTTCAAGACAAGGC	60	(AC)19	-	-	-	-		NA
CCOM27	F:CCTGCTCTCAAGTCTAGGCC<br>R:TGACTCCGTTTGCACAAGTG	60	(AC)20	-	-	-	-		NA
CCOM28	F:AGTCCCTGGTTCTCTCTGATAC<br>R:AGGCTTATCTGGAAGTGGTAGG	59	(AAT)11	2.000	0.454	0.234	52	82-91	P
CCOM29	F:TGGTGCTTGGTATATGGAGGG<br>R:GGAGCAGAGAAGGCATTGAG	60	(ATC)10	1.750	0.078	0.083	52	94-100	P
CCOM30	F:TGTCTCTACCTGCTTCCCTG<br>R:CAGCGTGCATGAGTGAAGAG	60	(AGC)9	-	-	-			M
CCOM31	F:ACTGCAACGAAGACCCAATG<br>R:TCACTCTGTCAACTGTGCCC	60	(AAT)9	3.000	0.476	0.464	53	84-96	P
CCOM32	F:GGATTGCTGACTCCCAAACC<br>R:ATCTTGGCTTGAGGTCTGGG	60	(AAC)8	-	-	-			M
CCOM33	F:ACATCAAGGACCTGGAGAGC<br>R:GAAATCGGCAGCTCACCTTC	60	(AGC)8	-	-	-			M
CCOM34	F:AGGGCTTGAATCCGTGTCC<br>R:ACAACAGTGAGAGGCCCG	60	(AAC)9	-	-	-	-		NA
CCOM35	F:AACGGGAGAGGTCACAACG<br>R:AGAGGTGGAAATTGACATGCG	60	(AAT)10	-	-	-	-		NA
CCOM36	F:TTAACGGTGCTCCCTGTGAC<br>R:TATTGCTGTGGTTGCTGTCC	60	(ATC)14	-	-	-	-		NA
CCOM37	F:TGACAGAGATGGTGATGACGG<br>R:GACCAGGGTACAGAGCTGAC	60	(ATC)9	-	-	-	-		NA
CCOM38	F:ATCTTGGCTTGAGGTCTGGG<br>R:GGATTGCTGACTCCCAAACC	60	(AAC)8	-	-	-			M
CCOM39	F:ACAAGAGTGAGAGGCCCATG<br>R:ACAGTGGGATGTCAAGCAC	59	(AAC)9	-	-	-	-		NA
CCOM40	F:AGGCGTACTCTCAACCACTG<br>R:TCCAGGCTTTCTACACCAGC	60	(AAG)13	-	-	-	-		NA
CCOM41	F:GACGGGATGCACATATTGGC<br>R:GGAGGAGGAATGTGCCAAAG	60	(AGC)8	1.250	0.017	0.018	53	90-93	P
CCOM42	F:GGTGCCCAAGAAAGAGTGTG<br>R:GCTTTCCTTGCTCCTCTGAC	60	(AGC)14	-	-	-	-		NA
CCOM43	F:CCACATGCTTTCCTTGCTCC<br>R:CCAAAGCAGAAGGAACGGC	60	(AGC)8	-	-	-	-		NA
CCOM44	F:ACAGTGAGAGGCCTGCATAC<br>R:TTCAAAGTTAGGAGCTGGGC	59	(AAC)8	2.000	0.128	0.103	53	105-114	P
CCOM45	F:GCATTTCTAGCAACCCAGGC<br>R:TATTCCTTCTGCCCTCACGC	60	(AGC)11	3.000	0.456	0.492	53	108-129	P
CCOM46	F:GAGAGGCCCGCGTAACAC<br>R:TGCCCATTCTAACTGGTGTG	60	(AC)12	-	-	-	-		NA
CCOM47	F:TCGAGAAGCTTGGGTGTGAG<br>R:CATTGACGCTCGGGTGAAC	60	(AC)11	-	-	-	-		NA
CCOM48	F:CCAACATGCCCGAATCAAAC<br>R:GGCGGTGGTGATTTGTATTG	58	(AC)18	-	-	-	-		NA
CCOM49	F:AGGCCTCAGAGACCTTACAG<br>R:AGCTGAAACTTGTCCATAGGG	59	(AG)11	3.000	0.539	0.504	53	95-99	P
CCOM50	F:GTCCAGACACCTCAAATGGAAG<br>R:AGACCAGGAATCCAAGTGACTC	60	(AC)12	1.500	0.043	0.046	53	95-97	P
CCOM51	F:GTTGATCTCACCACTGCCAG<br>R:GCTATGCCCAGAGACAGAC	59	(AC)10	2.000	0.489	0.480	53	95-97	P
CCOM52	F:TGTTCACAGCCATTCCAATCC<br>R:CCTTCTCCCTAAACGCACAC	59	(AG)17	5.000	0.634	0.687	53	87-107	P
CCOM53	F:GGCCACTCTGTATGCATGTC<br>R:AGCCTCTGTCTCCTATCATTGG	60	(AC)18	-	-	-	-		NA
CCOM54	F:GGCACCCTTATCATTCCAACAG<br>R:GCAGAAGTCCCTCCACCTC	60	(AC)18	5.000	0.675	0.801	51	93-103	P
CCOM55	F:ACACAAGACTTAGCTCCAAGG	59	(AC)18	-	-	-	-		NA

	R:CCCACCTCTCCTCCTTACC								
CCOM56	F:GGAAGGGCAAAGAAGAGGTG	60	(AC)10	1.750	0.094	0.065	52	97-99	P
	R:TTTCCCACTACCGACACTCC								
CCOM57	F:GTGTTCCCAAGAGAATGAGGC	60	(AG)18	-	-	-	-		NA
	R:AAGTGAGAAGGGCAGGAAGG								
CCOM58	F:CCCACCAGACCCAACTCTAAG	59	(AC)18	-	-	-	-		NA
	R:CACGAGCAACTTACTTTACTGG								
CCOM59	F:TCATTCCACAAACACGTATGGG	59	(AC)14	-	-	-	-		NA
	R:CTGAAAGAGAGCAGGCCC								
CCOM60	F:CACTGCCCACGTAGAACATG	60	(AC)13	3.000	0.239	0.190	48	91-97	P
	R:CATGAAGGACAGCAAGCCAG								
CCOM61	F:GGCATGAGTTGGAGAGTGTG	60	(AC)15	1.500	0.028	0.029	50	91-97	P
	R:TGTGAAATTCAGGTGGGAAAGC								
CCOM62	F:TCGTGACCTTCCCTAAACCC	59	(AC)16	-	-	-	-		NA
	R:CTTAACGCCATCAGAGTATCCC								
CCOM63	F:ACAACAGTGAGAGGCCCG	60	(AC)9	-	-	-	-		NA
	R:TAAGCTCTGGGATGTGTGGG								
CCOM64	F:GCCATGCCTCACCTCATTC	60	(AC)18	-	-	-	-		NA
	R:TGACCTGAAATGTTGGCACG								
CCOM65	F:AGGTGCCAGGACTTAGACAG	59	(AC)8	-	-	-			M
	R:TGATTAGGATGTAGGGCGGG								
CCOM66	F:GCAACATTTGGACCCAGGAC	60	(AC)13	-	-	-	-		NA
	R:AGGTGGTTAAGAGACAGGGTG								
CCOM67	F:GCACTGGAGCTGGGTTTC	59	(AC)18	-	-	-	-		NA
	R:TGTATCAACTTCCCTTCACACC								
CCOM68	F:GCCTAGATGTCTGCAGGATG	59	(AC)9	-	-	-	-		NA
	R:TATTGCCAGCGATGTGAGTG								
CCOM69	F:AGGAGTGTCAGGCAGTTGTG	60	(AC)10	1.250	0.017	0.018	53	95-97	P
	R:TGGAGCCTCTTACCTTTACCAC								
CCOM70	F:CCCTTCAACACCAATCTCTCC	60	(AG)10	-	-	-			M
	R:TGGACCGTGAACTTCAGCTC								

Table S4: Results of 10 independent runs of the Bayesian cluster analysis with 1 to 8 values of K parameter.

K	Est. Ln prob. of data	Mean value of Ln likelihood	Variance of Ln likelihood	K	Est. Ln prob. of data	Mean value of Ln likelihood	Variance of Ln likelihood
1	-1898.6	-1880.0	37.2	5	-1952.8	-1853.3	199.2
1	-1898.6	-1879.9	37.4	5	-1997.4	-1850.2	294.3
1	-1898.2	-1879.8	36.7	5	-1949.5	-1852.7	193.7
1	-1897.3	-1879.6	35.5	5	-1911.1	-1844.9	132.4
1	-1898.1	-1879.8	36.7	5	-1896.3	-1849.1	94.4
1	-1898.3	-1879.8	37.1	5	-1920.9	-1847.6	146.6
1	-1898.3	-1879.6	37.4	5	-1941.9	-1848.9	186.1
1	-1897.7	-1879.7	36.0	5	-1937.4	-1851.5	171.9
1	-1898.9	-1879.9	38.1	5	-1934.3	-1851.3	166.0
1	-1898.3	-1879.8	36.9	5	-1910.8	-1850.0	121.7
2	-1934.2	-1846.9	174.6	6	-1910.8	-1848.5	124.7
2	-1938.6	-1849.8	177.7	6	-1925.9	-1849.8	152.2
2	-1936.4	-1841.5	189.9	6	-1914.9	-1849.3	131.1
2	-1925.3	-1842.9	164.8	6	-1965.2	-1857.0	216.4
2	-1941.5	-1843.4	196.2	6	-1922.3	-1850.4	143.8
2	-1937.3	-1844.1	186.4	6	-1964.9	-1848.4	233.1
2	-1928.9	-1845.4	167.0	6	-1944.4	-1850.4	188.0
2	-1926.2	-1849.9	152.6	6	-1910.4	-1852.5	115.8
2	-1948.9	-1847.4	202.9	6	-1916.6	-1850.7	131.8
2	-1940.3	-1848.0	184.6	6	-1945.0	-1852.2	185.6
3	-1907.6	-1836.6	141.9	7	-1895.3	-1848.4	93.8
3	-1929.6	-1848.7	161.8	7	-1938.8	-1852.8	171.9
3	-1943.4	-1846.1	194.6	7	-1961.2	-1860.9	200.5
3	-1920.2	-1846.1	148.2	7	-1928.1	-1851.7	152.7
3	-1961.9	-1849.7	224.5	7	-1925.4	-1853.3	144.3
3	-1954.3	-1850.7	207.1	7	-1897.9	-1846.8	102.3
3	-1940.5	-1848.9	183.2	7	-1942.2	-1853.6	177.3
3	-1913.7	-1853.2	121.1	7	-1908.9	-1851.4	115.0
3	-1957.4	-1845.6	223.6	7	-1894.1	-1842.9	102.5
3	-1922.2	-1850.5	143.3	7	-1900.8	-1849.6	102.3
4	-1942.9	-1846.2	193.4	8	-1958.3	-1857.4	201.9
4	-1936.8	-1846.3	181.0	8	-1921.1	-1852.8	136.5
4	-1925.0	-1848.7	152.6	8	-1977.0	-1865.3	223.5
4	-1960.3	-1849.8	221.1	8	-1965.5	-1859.7	211.7
4	-1991.9	-1847.9	288.0	8	-1946.2	-1860.3	171.8
4	-1916.3	-1849.5	133.6	8	-1968.9	-1862.7	212.4
4	-1924.1	-1850.4	147.3	8	-1892.5	-1852.3	80.3
4	-1927.0	-1850.3	153.4	8	-1899.4	-1850.8	97.0
4	-1938.6	-1844.8	187.7	8	-1894.0	-1847.7	92.7
4	-1916.3	-1847.8	137.0	8	-1891.8	-1851.9	79.8

Table S5: Results of Evanno's method with 8 values of k parameter and ΔK. NA = not assigned.

K	Reps	Mean LnP(K)	Stdev LnP(K)	Ln'(K)	|Ln''(K)|	ΔK
1	10	-1898.230000	0.459589	NA	NA	NA
2	10	-1935.760000	7.358774	-37.530000	38.210000	5.192441
3	10	-1935.080000	19.192290	0.680000	3.520000	0.183407
4	10	-1937.920000	23.221724	-2.840000	5.520000	0.237708
5	10	-1935.240000	28.533575	2.680000	0.520000	0.018224
6	10	-1932.040000	21.302749	3.200000	9.570000	0.449238
7	10	-1919.270000	23.319808	12.770000	24.970000	1.070764
8	10	-1931.470000	35.278638	-12.200000	NA	NA

Table S6: Contemporary migration rate expressed as number of effective migrants among populations estimated with the program BAYESASS. Playa Unión (0), Bahía Camarones (1), Caleta Olivia (2), and Puerto Deseado (3).

	Mean (SE)		Mean (SE)		Mean (SE)		Mean (SE)
m[0][0]:	0.733 (0.054)	m[0][1]:	0.133 (0.067)	m[0][2]:	0.067 (0.055)	m[0][3]:	0.067 (0.054)
m[1][0]:	0.066 (0.054)	m[1][1]:	0.800 (0.067)	m[1][2]:	0.067 (0.055)	m[1][3]:	0.067 (0.054)
m[2][0]:	0.067 (0.055)	m[2][1]:	0.133 (0.067)	m[2][2]:	0.733 (0.054)	m[2][3]:	0.067 (0.054)
m[3][0]:	0.068 (0.055)	m[3][1]:	0.133 (0.067)	m[3][2]:	0.066 (0.054)	m[3][3]:	0.733 (0.055)

Migration rates are expressed as follows: m[X][Y], probability that an individual from population Y comes from population X.

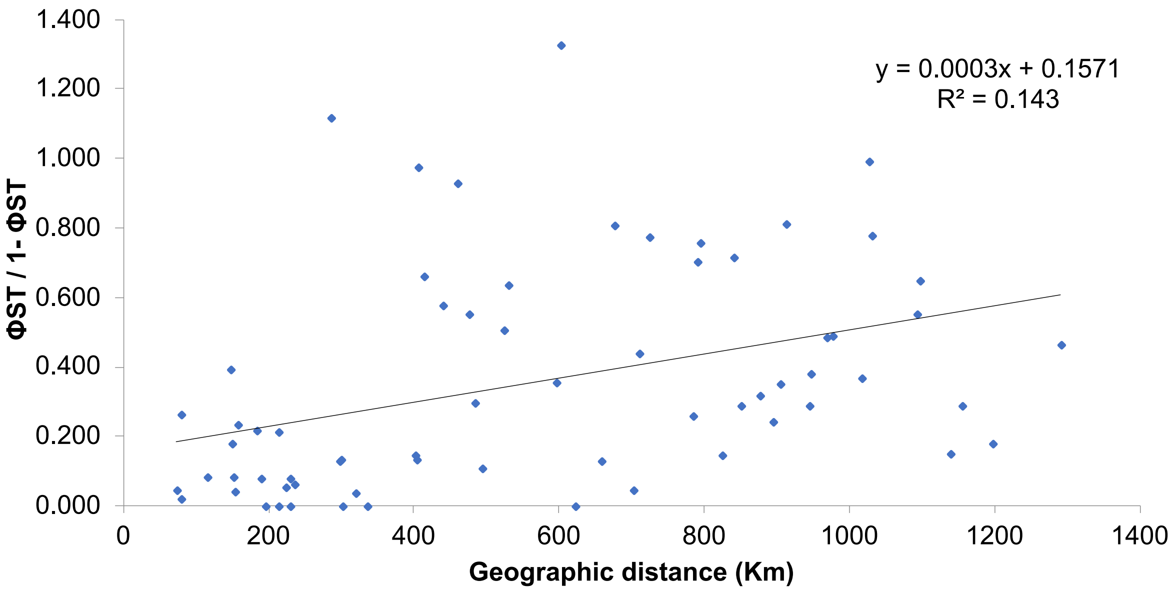

Figure S1: Scatter plot of Mantel test for isolation by distance of *Cephalorhynchus commersonii* based on mitochondrial DNA control region sequences from 12 sampling sites (N = 308, 423 bp).

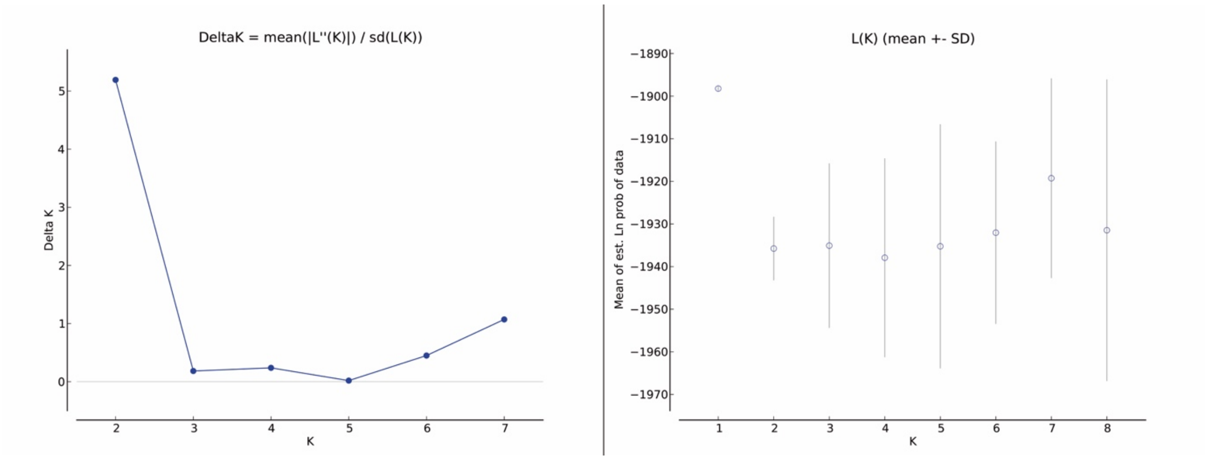

Figure S2: Evanno and Ln Probability plots for STRUCTURE analysis including all sampling location and generated using STRUCTURE HARVESTER (Earl and vonHoldt 2012).

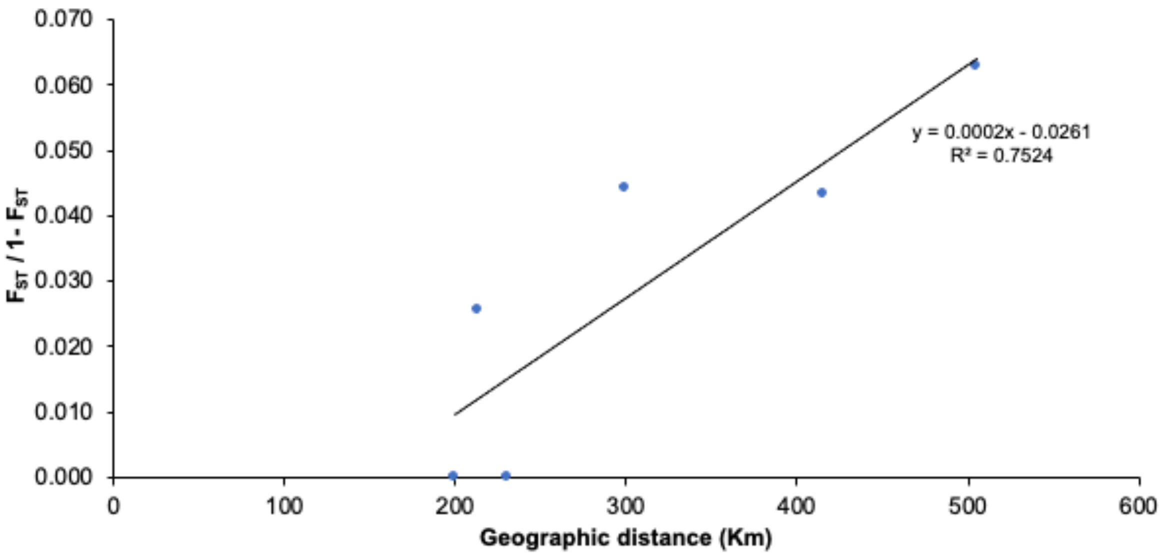

Figure S3: Scatter plot of Mantel test for isolation by distance of *Cephalorhynchus commersonii* (N = 50) based on 28 microsatellite loci from 4 sampling sites.

References

Earl, D. A. and vonHoldt, B. M. 2012. STRUCTURE HARVESTER: a website and program for visualizing STRUCTURE output and implementing the Evanno method. Conserv. Genet. Res. 4(2): 359-361.
