# Supplementary material for: Commerson’s dolphin population structure: evidence for female phylopatry and male dispersal
Source: Sci Rep. 2022 Dec 23;12:22219. doi: 10.1038/s41598-022-26192-0 (PMC9789068; doi:10.1038/s41598-022-26192-0)
Supplement: Supplementary file 2 — Supplementary Information 2. [file 41598_2022_26192_MOESM2_ESM.pdf]

COMMERSON`S DOLPHIN POPULATION STRUCTURE: EVIDENCE FOR FEMALE PHYLOPATRY AND MALE DISPERSAL

Cristian Alberto Durante, Rocio Loizaga, Gregory R. McCracken, Enrique Alberto Crespo, Daniel E. Ruzzante.

Supplementary material 2

Table S1: AMOVA statistics for mitochondrial DNA control region sequences from Commerson's dolphin (*Cephalorhynchus commersonii*) using data subset without highly relatedness individuals (n = 42).

Analysis	Source of variation	d.f.	Sum of squares	Variance components	Percentage of variation	Statistics	p-value (α=0.05)
Haplotype frequencies	Among populations	3	3.56	0.085	20.59	F_{ST} = 0.206	<0.001
	Within populations	38	12.47	0.328	79.41		
	Total	41	16.02	0.413			
Distance method: Kimura 2-parameter	Among populations	3	6.16	0.104	9.30	Φ_{ST} = 0.093	0.024
	Within populations	38	38.38	1.010	90.70		
	Total	41	44.54	1.114			

Table S2: Genetic differentiation among pairwise populations of Commerson's dolphin (*Cephalorhynchus commersonii*) using the data subset without relatedness individuals in: **a-** mitochondrial DNA control region (423 bp) (F_{ST} values are reported below the diagonal, whereas Φ_{ST} values are reported above the diagonal). **b-** 28 microsatellites loci. Significant values (α = 0.05) are in bold font. PU = Playa Unión, BC = Bahía Camarones, CO = Caleta Olivia, PD = Puerto Deseado.

		Distance method: Kimura 2-parameter			
**a-**		PU	BC	CO	PD
Computing conventional F-Statistics from haplotype frequencies	PU		0.03604	0.10980	0.11841
	BC	0.02111		-0.02740	**0.17621**
	CO	0.09153	-0.02870		**0.24865**
	PD	**0.30421**	**0.40590**	**0.43000**	
**b-**					
Number of different alleles	BC	0.02145			
	CO	0.01604	-0.00849		
	PD	0.02884	0.01230	-0.03053	

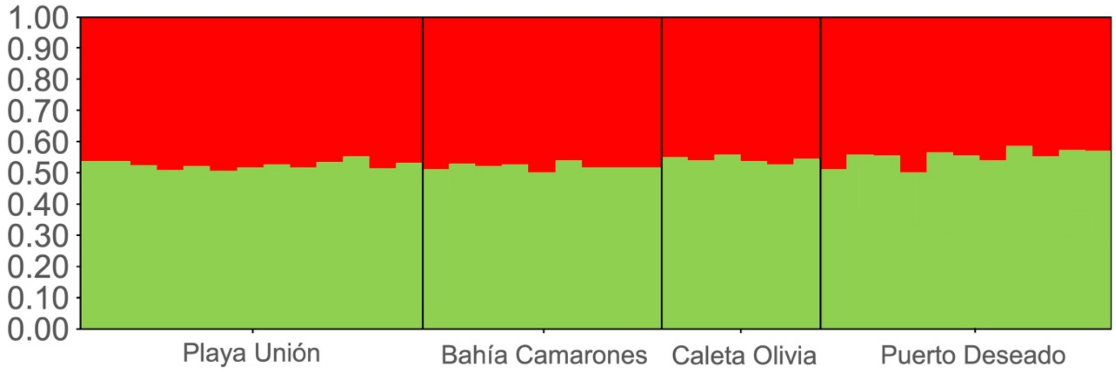

Fig. S1: Hierarchical STRUCTURE analysis of Commerson's dolphin (*Cephalorhynchus commersonii*) from the four sampling regions using the subset without relatedness individuals (n=39) and 28 microsatellite loci. Each individual is represented by a vertical line, which is partitioned into K colored segments, where each colored segment length is proportional to the individual's estimate membership coefficient.

Table S3: Results of 10 independent runs of the Bayesian cluster analysis with 1 to 8 values of K parameter.

K	Est. Ln prob. of data	Mean value of Ln likelihood	Variance of Ln likelihood	K	Est. Ln prob. of data	Mean value of Ln likelihood	Variance of Ln likelihood
1	-1479.4	-1463	32.8	5	-1485.6	-1460.3	50.7
1	-1479.7	-1462.9	33.5	5	-1483.6	-1460.3	46.5
1	-1479.7	-1463	33.4	5	-1479.3	-1461.6	35.4
1	-1479.3	-1463.1	32.3	5	-1480.7	-1461.3	38.8
1	-1479.6	-1463	33.3	5	-1480.8	-1461	39.6
1	-1481.7	-1463.4	36.5	5	-1480.2	-1461.1	38.1
1	-1479.7	-1463	33.4	5	-1487.8	-1460.3	55.2
1	-1479.6	-1463	33.2	5	-1482.7	-1460.6	44.3
1	-1480	-1463.1	33.7	5	-1483	-1460.8	44.5
1	-1479.7	-1462.9	33.7	5	-1482.7	-1460.7	44.1
2	-1486.6	-1460.6	51.9	6	-1481.4	-1460.9	40.9
2	-1486.5	-1459.8	53.4	6	-1482.9	-1461.1	43.4
2	-1482.6	-1460.9	43.3	6	-1483.2	-1461.8	42.8
2	-1484.1	-1461.4	45.3	6	-1481.6	-1461	41.2
2	-1485.7	-1461.4	48.6	6	-1485.8	-1459.9	51.9
2	-1487	-1460.3	53.4	6	-1482.7	-1461.2	43
2	-1486.2	-1461.1	50.2	6	-1479.9	-1461.8	36.2
2	-1484.6	-1461.3	46.7	6	-1482.6	-1460.9	43.4
2	-1488.6	-1460.3	56.7	6	-1480.3	-1461.7	37.2
2	-1478.8	-1461.9	33.8	6	-1482.9	-1460.9	43.9
3	-1483.9	-1460.3	47.1	7	-1480.8	-1461.4	38.9
3	-1484.8	-1460.7	48.1	7	-1481.2	-1461.5	39.5
3	-1484	-1460.9	46.2	7	-1480.7	-1461.4	38.7
3	-1481.4	-1461.7	39.4	7	-1480.9	-1461.4	39
3	-1487.6	-1460.4	54.4	7	-1480.6	-1462	37.2
3	-1483.7	-1461.2	45	7	-1481.9	-1462.2	39.2
3	-1485.4	-1460.1	50.6	7	-1479	-1461.7	34.5
3	-1484.3	-1461.9	44.8	7	-1483.2	-1462.5	41.4
3	-1483.9	-1460.8	46.3	7	-1481.7	-1461.8	39.9
3	-1486.7	-1460.8	51.7	7	-1484.1	-1459.8	48.5
4	-1483.4	-1460.7	45.3	8	-1480.8	-1462	37.7
4	-1481	-1461.5	39.1	8	-1483.5	-1461.1	44.9
4	-1484.8	-1460.1	49.6	8	-1479.7	-1461.6	36.2
4	-1483.8	-1460.6	46.3	8	-1481.3	-1461.2	40.2
4	-1481.1	-1461	40.2	8	-1480.1	-1462	36.3
4	-1483.5	-1460.5	45.9	8	-1481.2	-1462.8	36.8
4	-1482.5	-1461.3	42.4	8	-1482.4	-1461.3	42.3
4	-1482.6	-1461	43.2	8	-1480	-1461.7	36.7
4	-1486.6	-1459.6	54	8	-1481.9	-1460.6	42.6
4	-1480.1	-1461.4	37.4	8	-1484.6	-1460.1	49

Table S4: Results of Evanno's method with 8 values of k parameter and ΔK. NA = not assigned.

K	Reps	Mean LnP(K)	Stdev LnP(K)	Ln'(K)	|Ln''(K)|	ΔK
1	10	-1479.840000	0.680196	NA	NA	NA
2	10	-1485.070000	2.765281	-5.230000	5.730000	2.072122
3	10	-1484.570000	1.717912	0.500000	1.130000	0.657775
4	10	-1482.940000	1.933448	1.630000	1.330000	0.687890
5	10	-1482.640000	2.597092	0.300000	0.010000	0.003850
6	10	-1482.330000	1.669364	0.310000	0.610000	0.365409
7	10	-1481.410000	1.430190	0.920000	1.060000	0.741160
8	10	-1481.550000	1.585525	-0.140000	NA	NA

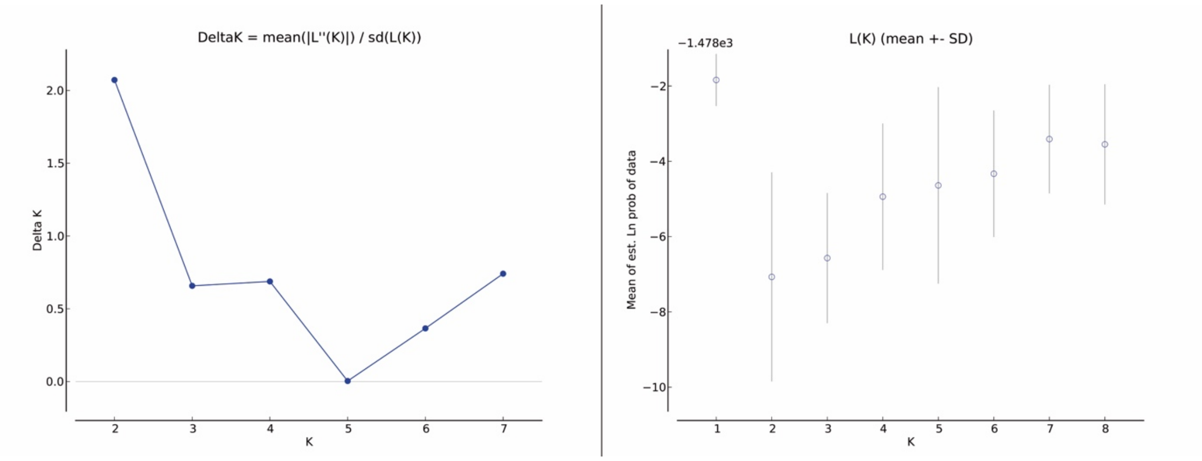

Figure S2: Evanno and Ln Probability plots for STRUCTURE analysis for the data subset without relatedness individuals and generated using STRUCTURE HARVESTER (Earl and vonHoldt 2012).

References

Earl, D. A. and vonHoldt, B. M. 2012. STRUCTURE HARVESTER: a website and program for visualizing STRUCTURE output and implementing the Evanno method. Conserv. Genet. Res. 4(2): 359-361.
